# The SIX5 Protein in *Fusarium oxysporum* f. sp. *cepae* Acts as an Avirulence Effector toward Shallot (*Allium cepa* L. Aggregatum Group)

**DOI:** 10.3390/microorganisms11122861

**Published:** 2023-11-26

**Authors:** Kosei Sakane, Masaaki Kunimoto, Kazuki Furumoto, Masayoshi Shigyo, Kazunori Sasaki, Shin-ichi Ito

**Affiliations:** 1 The United Graduate School of Agricultural Sciences, Tottori University, Tottori 680-8553, Japan; necchange999@outlook.jp; 2 Graduate School of Sciences and Technology for Innovation, Yamaguchi University, Yamaguchi 753-8515, Japan; w019gd@yamaguchi-u.ac.jp (M.K.); g030vh@yamaguchi-u.ac.jp (K.F.); shigyo@yamaguchi-u.ac.jp (M.S.); 3 Research Center for Thermotolerant Microbial Resources (RCTMR), Yamaguchi University, Yamaguchi 753-8515, Japan

**Keywords:** *Fusarium oxysporum* f. sp. *cepae*, shallot, virulence effector, SIX5

## Abstract

*Fusarium oxysporum* f. sp. *cepae* (*Foc*) causes basal rot disease in *Allium* species, including onions (*Allium cepa* L.) and shallots (*A. cepa* L. Aggregatum group). Among *Allium* species, shallots can be crossbred with onions and are relatively more resistant to *Foc* than onions. Thus, shallots are considered a potential disease-resistant resource for onions. However, the mechanisms underlying the molecular interactions between shallots and *Foc* remain unclear. This study demonstrated that SIX5, an effector derived from *Foc* (FocSIX5), acts as an avirulence effector in shallots. We achieved this by generating a *FocSIX5* gene knockout mutant in *Foc,* for which experiments which revealed that it caused more severe wilt symptoms in *Foc*-resistant shallots than the wild-type *Foc* and *FocSIX5* gene complementation mutants. Moreover, we demonstrated that a single amino acid substitution (R67K) in FocSIX5 was insufficient to overcome shallot resistance to *Foc*.

## 1. Introduction

The *Fusarium oxysporum* species complex is a ubiquitous, soil-borne, and plant-pathogenic fungus with a wide host range comprising more than 120 species. Therefore, it has recently been recognized as the fifth most important plant pathogen [1,2]. Based on host specificity, *F. oxysporum* species are generally distinguished as “*formae speciales*” [3,4], among which *F. oxysporum* f. sp. *cepae* (*Foc*) has been identified as the causative agent of Fusarium basal rot in onions (*Allium cepa* L.). Globally, onion production is threatened by *Foc* [5,6,7,8]. Hence, understanding plant defense systems against *Foc* and *Foc*-infective mechanisms is important for achieving sustainable onion production. Moreover, durable genetic resources are desirable for breeding disease-resistant onions.

Shallot (*A. cepa* L. Aggregatum group) is an annual herbaceous plant belonging to the family Amaryllidaceae, which is widely used as a condiment in Southeast Asian countries. It contains antimicrobial compounds [9,10] and is highly resistant to pathogens, including *F. oxysporum* [11,12]. Shallots are genomically compatible and can be crossbred with onion plants [13]. Therefore, shallots are considered a versatile breeding resource for onions [14].

Plants have evolved multilayered barrier systems to protect themselves against pathogens. Plants recognize pathogen-associated molecular patterns (PAMPs) using plant cell-surface-localized pattern-recognition receptors (PRR) to induce pattern-triggered immunity (PTI). To impede the PTI response, pathogens secrete proteins with signal peptide motifs that translocate into the host tissue to manipulate the PTI response, known as effector-triggered susceptibility (ETS) [15,16]. Subsequently, plants recognize pathogenic effector molecules using resistance proteins to trigger a robust immune response called effector-triggered immunity (ETI) [17]. In *F. oxysporum*, the genetic concept of F. oxysporum is well characterized in tomato plants and *F. oxysporum* f. sp. *lycopersici* (*Fol*). Reportedly, *Fol* secretes the SIX (secreted in the xylem) effector protein into the xylem to facilitate colonization during infection. To date, 14 SIX proteins have been identified in *Fol*-infecting tomato xylem sap [18,19], of which SIX1(AVR3), SIX3 (AVR2)*,* SIX5, and SIX6 are required for the virulence of tomato plants. Conversely, SIX1(AVR3), SIX3 (AVR2)-SIX5, and SIX4 (AVR1) are avirulence effectors that activate the immunity of tomato plants, mediated by *I-3* (SRLK-type), *I-2* (CC-type), and *I* genes, respectively [20,21,22,23,24,25]. These resistance genes have been introduced into commercial tomato cultivars for stable and effective production [26]. However, *Fol* adopts several strategies to evade the tomato immune system. The *Fol* race 2 strain completely lost the *SIX4* (*AVR1*) gene to avoid the *I* gene-derived immunity [27], whereas the *Fol* race 3 strain had a single amino acid substitution in its SIX3 (AVR2) sequence to escape *I-2*-derived immunity [22].

In the *Foc* genome, the sequences of a few *SIX* genes (*SIX3*, *SIX5*, *SIX7*, *SIX9*, *SIX10*, *SIX12*, and *SIX14*) are conserved [8,28]. Among these *SIX* genes, *SIX5* (*FocSIX5*) is the most highly upregulated during *Foc* infection in onions [28]; however, its virulence in *Foc* has not yet been elucidated. In this study, we generated *FocSIX5* gene-modified mutants of *Foc* and conducted pathogenicity tests on both *Foc*-susceptible onions and *Foc*-resistant shallots using *FocSIX5* gene-modified mutants to investigate the function of FocSIX5.

## 2. Materials and Methods

### 2.1. Plant Material and Fungal Strain

Shallots (*A. cepa* L. Aggregatum group) cv. Chiang Mai (SAMD00027216) [13] and onion cultivars “Kitamomiji 2000” (Shippou Co., Ltd., Kagawa, Japan) and “Tarzan” (Shippou Co., Ltd.) were used for this study. The *Foc*_TA isolate from the fungal strain *F. oxysporum* f. sp. *cepae* (*Foc*) was collected from a diseased onion bulb in Hokkaido, Japan [6].

### 2.2. Pathogenicity Test toward Onion and Shallot Plants

For the pathogenicity test on onion bulbs, onion cv. “Kitamomiji 2000” was used as the host. *Foc*_TA was grown on potato dextrose agar (PDA) medium, and the medium was incubated in a growth chamber with a temperature of 25 °C for 5 d. Onion bulbs were surface-sterilized with 0.05% NaOCl for 3 min; then, the central basal part of the sterilized onion bulbs was hollowed out with a 5 mm cork borer. The edge of the colony was then hollowed out with the 5 mm cork borer and embedded in the hollowed basal part of the sterilized onion bulbs. A plane PDA medium plug was embedded in the basal tissue of the hollowed-out onion as a control. The inoculated onion bulb was placed inside a plastic bag with a wet paper towel and incubated in a temperature-controlled room with a temperature of 25 °C. After 4 weeks, the symptoms of the inoculated onion bulb were observed. The symptomatic areas, including mycelia and brown discoloration, were manually captured and estimated from the photographs using ImageJ1 software [29]. All the tests were conducted with at least three samples per iteration. All experiments were repeated at least twice.

For the pathogenicity test of shallot and onion seedlings, shallot cv. Chiang Mai [13] and onion cv. “Tarzan” were used. Fungal isolates were cultured in potato dextrose broth for seven days in a growth chamber with a temperature of 25 °C, with shaking at 120 rpm, and the cultures were filtered through three layers of sterilized gauze to collect spores for inoculation. The spores were then collected and rinsed once with sterile water. The number of spores in the suspension was determined using a hemocytometer and adjusted to a concentration of 1 × 10^6^ spores/mL. Shallot bulbs and onion seeds were sown in plastic pots filled with a mixture of sand and compost at a ratio of 4:1. The pots were incubated for seven days in a temperature-controlled room with a temperature of 25 °C and a 16:8 light–dark cycle, after which the seedlings were uprooted and the central portion of the root was excised. The cut portion of the root was dipped into the prepared spore suspension and sterilized water (as a control) for 1 h. Subsequently, the inoculated seedlings were transplanted into plastic pots containing the same soil mixture. The pots were then placed in a temperature-controlled room with a temperature of 25 °C and 16:8 light-dark cycle.

The shallot disease index was scored five weeks postinoculation, as described previously [30], with slight modifications: 0, no chlorosis; 1, necrosis on the tip of the leaf; 2, leaf curving with a pale green or yellowish color; 3, leaf curving and drying out; and 4, leaf death. The disease index was evaluated for each leaf, and an average disease index was calculated for each plant using the following equation: disease index = (4 × n [number of leaf deaths] + 3 × n [number of leaves curving and drying out] + 2 × n [number of leaves curving with a pale green or yellowish color] + 1 × n [number of necrosis on the tip of the leaf] + 0 × n [leaf number with no chlorosis])/total number of evaluated leaves. The biomass of all the shallot plants was also measured. The pathogenicity test was conducted at least twice with at least three seedlings per iteration.

### 2.3. RNA Extraction and Quantitative Reverse-Transcriptase Polymerase Chain Reaction (qRT-PCR)

To conduct the quantitative reverse-transcription polymerase chain reaction (qRT-PCR), RNA was extracted from onion and shallot roots inoculated with *Foc*_TA at 3, 7, and 14 days postinoculation (dpi). Total RNA was extracted from three independent onion and shallot root samples using Sepasol-RNA I Super G (Nacalai Tesque Inc., Kyoto, Japan). For reverse transcription, 500 ng of total RNA was used in a 10 µL reaction volume with the ReverTra Ace qPCR RT Master Mix with gDNA Remover (Toyobo), following the manufacturer’s instructions. The resulting cDNA was diluted (1:1), and 1 µL of the diluted cDNA was used as a template in a 20 µL total volume of THUNDERBIRD SYBR qPCR Mix (Toyobo). The relative amounts of *FocSIX5* gene transcripts were calculated and normalized to that of the *EF-1α* gene. Real-time quantitative PCR was performed using a 7300 system (Applied Biosystems, Foster City, CA, USA).

### 2.4. Sequence Alignment and Prediction of Signal Peptide

Amino acid sequences were aligned using the Clustal 2.1 [31]. Signal peptides were predicted using SignalP-5.0 [32]. Sequence data of SIX5 were obtained from the NCBI database: (Fol4287 (XP_018257286), FUS2 (ALQ80804), Fus062 (QMX85381), Fus125 (QMX85382), Fus127 (QMX85383), Fus129 (QMX85384), A21 (ALQ80805), Fox129 (UVW62045), and AP117 (LC731005)).

### 2.5. Generation of Gene Knockout and Complementation Constructs

A fusion PCR strategy was used to generate a gene knockout construct [33]. The 5′ and 3′ flanking regions of the *FocSIX5* gene were amplified using SIX5-split-F1/F2 and SIX5-split-F3/F4 primer sets, respectively (Table 1). The *hph* (Hygromycin B resistance) cassette was amplified from the pHRC vector using the M13F/M13R primer set. The three obtained amplicons were fused using fusion PCR using the SIX5-split-F1/F4 primer set.

To generate a gene complementation mutant, a DNA construct containing an open reading frame (ORF) upstream and downstream of the *FocSIX5* gene was amplified using the SIX5-split-F1/F4 primer set. A geneticin-resistance gene cassette was amplified from the pII99 plasmid [36].

### 2.6. Protoplast Preparation

Fungal protoplasts were prepared as previously described [37] with slight modifications. The enzyme solution contained 10 mg/mL lysing enzymes (Sigma-Aldrich, St. Louis, MO, USA) and 4 mg/mL yatalase (Takara Bio, Shiga, Japan). The protoplast concentration was adjusted to 1.0 × 10^8^ cells/mL in STC buffer (1.2 M sorbitol, 50 mM CaCl2, 10 mM Tris-HCl, pH 7.4).

### 2.7. Fungal Transformation

Polyethylene glycol (PEG)-mediated fungal transformation was performed to generate gene knockout and complementation mutants. For the gene knockout mutant, 20 µg of the gene knockout construct and 920 µL 60% PEG solution were added to the protoplast suspension [38]. Hygromycin B resistance mutants were selected as candidates for *FocSIX5* gene knockout mutant and incubated on PDA-containing hygromycin B (100 µg/mL). The DNA of the candidate of the *FocSIX5* gene knockout mutant was extracted using a simple extraction method described previously [39]. *FocSIX5* knockout was verified via PCR using Quick Taq HS (Toyobo, Osaka, Japan), following the manufacturer’s instructions, with the SIX5-C-F/SIX5-C-R and SIX5-split-F1/SIX5-split-F4 primer sets. Furthermore, *FocSIX5* knockout was verified using Southern blot analysis. In brief, the downstream region of the *FocSIX5* gene was amplified using the SIX5-split-F3/SIX5-split-F4 primer set, and digoxigenin was labeled as a hybridization probe. The total DNA was extracted from the mycelia of wild-type *Foc*_TA and candidate *FocSIX5* knockout mutants cultured for 5 days. Thereafter, 10 µg of total DNA of the wild-type *Foc*_TA and candidates for *FocSIX5* gene knockout mutants was digested using the *Eco*RV restriction enzyme and, after blotting, hybridized using the hybridization probe. The digoxigenin-labeled probe was detected using a CDP-Star^TM^ detection reagent (Roche Diagnostics Deutschland GmbH, Mannheim, Germany) according to the manufacturer’s instructions. Finally, two *FocSIX5* gene knockout mutants (ΔSIX5-1 and ΔSIX5-2) were generated and used for further investigation.

To obtain the complementation mutants, 10 µg of the complementation construct and 10 µg of the geneticin-resistance cassette were co-transformed into fungal protoplasts. Geneticin-resistance mutants were selected as candidates of *FocSIX5* gene complementation mutant and incubated on PDA-containing G418 (100 µg/mL). DNA of the candidate of *FocSIX5* gene complementation mutant was extracted [39], and *FocSIX5* gene complementation was verified through PCR using the SIX5-C-F/SIX5-C-R and SIX5-split-F1/SIX5-split-F4 primer sets. Consequently, two *FocSIX5* gene complementation mutants (ΔSIX5-1 + SIX5 [Δ-1 + SIX5] and ΔSIX5-2 + SIX5 [Δ-2 + SIX5]) and two *FocSIX5* gene complementation mutants with *FocSIX5* gene variant G200A SNP (ΔSIX5-2 + SIX5^R67K^-1 [Δ-2 + SIX5^R67K^-1] and ΔSIX5-2 + SIX5^R67K^-2 [Δ-2 + SIX5^R67K^-2]) were generated and used for further investigation.

### 2.8. Vegetative Growth Assays

Wild-type *Foc*_TA and the gene knockout and gene complementation mutants were cultured on PDA in a growth chamber with a temperature of 25 °C for five days. The colony edge was collected using a 5 mm cork bore, and the mycelia plug were placed in the center of PDA plates and incubated in a growth chamber with a temperature of 25 °C for five days. The colony diameters were measured. All the tests were conducted with at least three samples per iteration. All experiments were repeated at least twice.

### 2.9. Statistical Analysis

The experimental data are presented as the mean and standard error. The statistical significance of the differences between the mean values was determined using the Student’s *t*-test or one-way analysis of variance with post hoc ANOVA and post hoc Tukey HSD test.

## 3. Results

### 3.1. Confirmation of Pathogenicity of Foc_TA toward Onion and Shallot Plants

**Figure 1:**
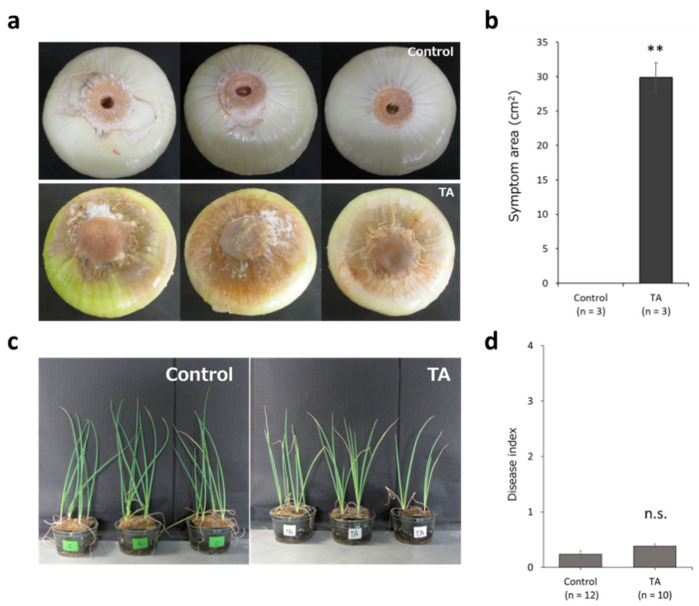
Results of the pathogenicity test of *Foc*_TA toward onion and shallot plants. (**a**) Symptoms of non-inoculated and *Foc*_TA-inoculated onions. (**b**) Symptom area on inoculated onion bulb. n represents sample size. Asterisks indicate a significant difference (** *p* < 0.01) compared to the control using a Student’s *t*-test. (**c**) Symptoms of non-inoculated and *Foc*_TA–inoculated shallots. (**d**) Average disease index of shallot plants inoculated with wild-type *Foc*_TA five weeks after inoculation. n represents the sample size. n.s. denotes “not significant” compared to control using a Student’s *t*-test. All data are presented as mean and standard error.

Inoculation tests were performed to confirm the pathogenicity of *Foc*_TA on onion and shallot plants. These results showed that *Foc*_TA caused severe basal rot disease in onion bulbs. However, shallot seedlings inoculated with *Foc*_TA exhibited only slight necrosis of the leaf tip. (Figure 1). All onion seedlings inoculated with *Foc*_TA exhibited severe leaf-death symptoms.

### 3.2. Expression of FocSIX5 Gene in Onion and Shallot Plants during Infection

*FocSIX5* is drastically upregulated during onion infection [28]. Therefore, qRT-PCR was performed to examine the expression of *FocSIX5* in *Foc*_TA during onion and shallot infections. qRT-PCR showed that the *FocSIX5* gene was expressed in shallot and onion roots inoculated with *Foc*_TA (Figure S1).

### 3.3. Characterization of FocSIX5

**Figure 2:**
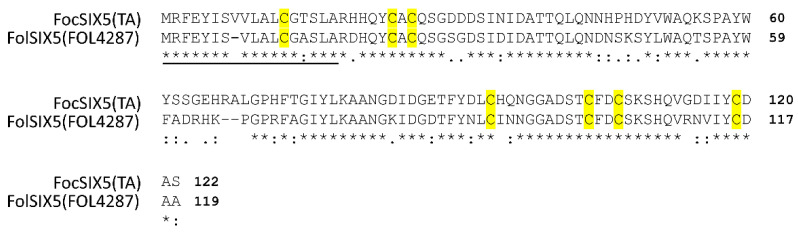
Alignment of the amino acid sequences of FocSIX5 and FolSIX5. The underline in the sequence alignment shows the signal peptides predicted using SignalP5.0. The yellow boxes indicate cysteine residues in the amino acid sequences. The asterisks (*) indicate identical amino acids.

We compared the amino acid sequences of FocSIX5 and SIX5 in FoL 4287 (FolSIX5, Accession no. XP_018257286). FocSIX5 was predicted to be a secretory peptide harboring seven cysteine residues, encoding 122 amino acids with 13.4 a (Accession of. LC730887). According to the BLAST analysis, FocSIX5 was 74% similar to FolSIX5, and the signal peptide of FocSIX5/FolSIX5 was predicted to be cleaved at the alanine residue. The cysteine residues were conserved between FocSIX5 and FolSIX5 (Figure 2).

### 3.4. Generation of a FocSIX5 Gene-Modified Mutant

To clarify the involvement of FocSIX5 in pathogenicity, we generated a *FocSIX5* knockout mutant via marker-exchange homologous recombination with a hygromycin B resistance gene (*hph*) cassette (Figure S2). *FocSIX5* knockout mutants were complemented by reintroducing the *FocSIX5* construct and a geneticin-resistance cassette. Subsequently, gene modification was verified using a polymerase chain reaction (PCR) using the designated primer set and using Southern blot analysis (Figures S3 and S4).

### 3.5. Mycelial Growth of FocSIX5 Gene-Modified Mutant

To investigate the effects of *FocSIX5* modification on phenotypic traits, the fungal development in wild-type *Foc*_TA, *FocSIX5* knockout, and *FocSIX5* complementation mutants was evaluated. No marked differences were observed in mycelial growth among the wild-type *Foc*_TA, *FocSIX5* knockout, or *FocSIX5* complementation mutants (Figure S5).

### 3.6. Effect of the FocSIX5 Gene Modification on Pathogenicity toward Onion and Shallot

**Figure 3:**
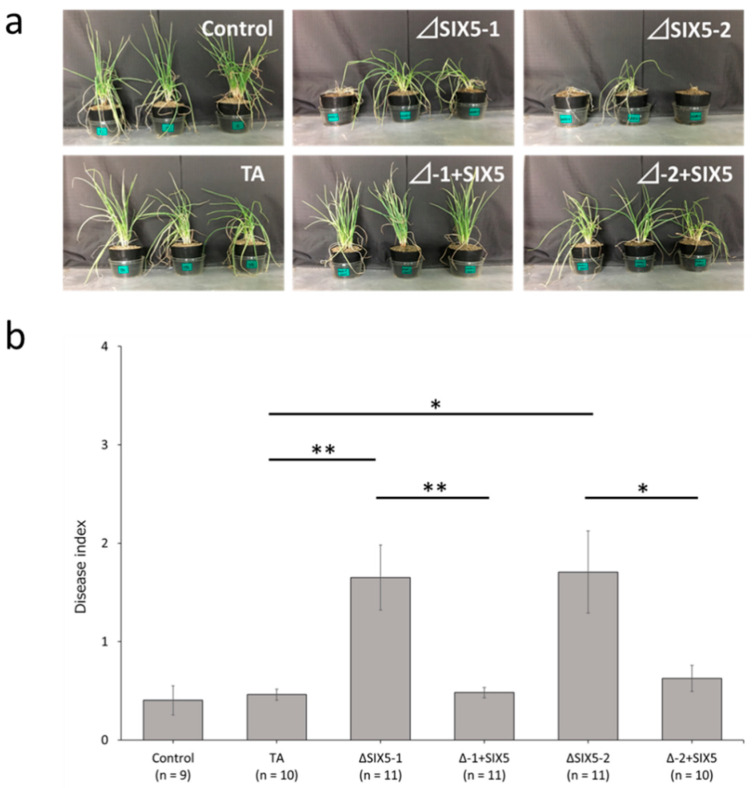
Results of the pathogenicity test toward shallot plants with *FocSIX5* gene knockout and gene complementation mutant. (**a**) Photographs of representative shallot plants inoculated with wild-type *Foc*_TA, *FocSIX5* gene knockout (ΔSIX5-1 and ΔSIX5-2), and *FocSIX5* gene complementation mutants (ΔSIX5-1 + SIX5 [Δ-1 + SIX5] and ΔSIX5-2 + SIX5 [Δ-2 + SIX5]) five weeks after inoculation. (**b**) Average disease index of shallot plants inoculated with wild-type *Foc*_TA, *FocSIX5* gene knockout (ΔSIX5-1 and ΔSIX5-2), and *FocSIX5* gene complementation mutants (ΔSIX5-1 + SIX5 [Δ-1 + SIX5] and ΔSIX5-2 + SIX5 [Δ-2 + SIX5]) five weeks after inoculation. Results of at least two experiments were combined. Asterisks indicate a significant difference (** *p* < 0.01, * *p* < 0.05) evaluated using a Student’s *t*-test. n represents sample size. Data are presented as mean and standard error.

*FolSIX5* acts as both a virulence and an avirulence gene in the *Fol*–tomato pathosystem [23]. Thus, onion bulbs were inoculated with *FocSIX5* gene knockout (ΔSIX5-1 and ΔSIX5-2) and *FocSIX5* gene complementation mutants (ΔSIX5-1 + SIX5 [Δ-1 + SIX5] and ΔSIX5-2 + SIX5 [Δ-2 + SIX5]) to investigate whether *FocSIX5* gene is related to pathogenicity toward onion. *FocSIX5* knockout mutants did not remarkably compromise virulence but slightly decreased the symptom area on the onion bulb compared to the wild-type *Foc*_TA and *FocSIX5* complementation mutants (Figure S6). Pathogenicity tests for shallots were performed using wild-type *Foc*_TA, *FocSIX5* knockout, and *FocSIX5* complementation mutants. The shallot plants used in this study exhibited a highly *Foc*-resistant phenotype; therefore, shallot plants inoculated with wild-type *Foc*_TA did not exhibit severe wilting symptoms. However, shallot plants inoculated with the *FocSIX5* knockout mutant showed more severe wilting than those inoculated with the wild-type *Foc*_TA, and the biomass of shallot plants inoculated with *FocSIX5* knockout mutant was consistently lower than that of shallot plants inoculated with the wild-type *Foc*_TA and *FocSIX5* knockout mutants (Figure S7). Moreover, no severe wilt symptoms were observed in shallot plants inoculated with the *FocSIX5* complementation mutants, demonstrating that FocSIX5 acts as an intact avirulence effector in shallots (Figure 3).

### 3.7. Effect of G200A Mutation on the Pathogenicity toward Shallot

**Figure 4:**
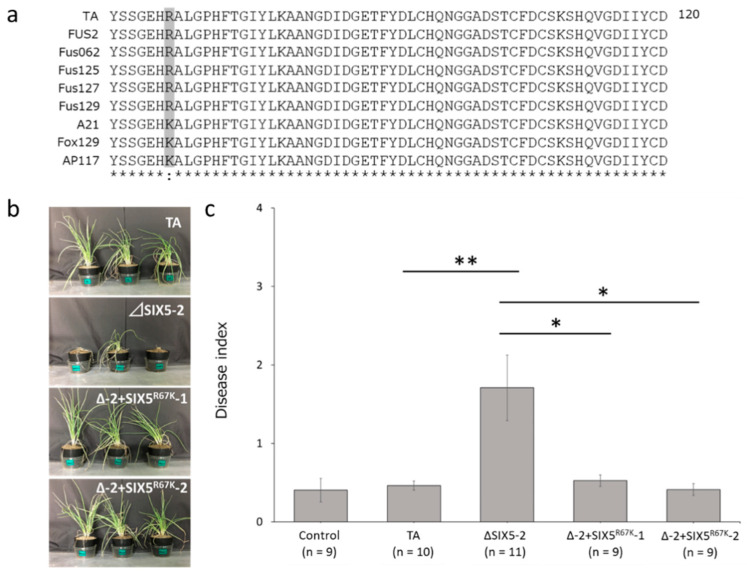
Results of the pathogenicity test after single amino acid substitution R67K in the FocSIX5 sequences. (**a**) Alignment of amino acid sequences of FocSIX5. The gray box shows sequence variation in FocSIX5 among *Foc* strains. (**b**) Photographs of representative shallot plants inoculated via *FocSIX5* gene knockout (ΔSIX5-2) and gene complementation mutant with *FocSIX5* gene variant G200A SNP (ΔSIX5-2 + SIX5^R67K^-1 [Δ-2 + SIX5^R67K^-1] and ΔSIX5-2 + SIX5^R67K^-2 [Δ-2 + SIX5^R67K^-2]) five weeks after inoculation. (**c**) Average disease index of shallot plants inoculated withthe *FocSIX5* gene knockout mutant (ΔSIX5-2) and gene complementation mutant with *FocSIX5* gene variant G200A SNP (ΔSIX5-2 + SIX5^R67K^-1 [Δ-2 + SIX5^R67K^-1] and ΔSIX5-2 + SIX5^R67K^-2 [Δ-2 + SIX5^R67K^-2]) five weeks after inoculation. Results of at least two experiments were combined. Asterisks indicate a significant difference (** *p* < 0.01, * *p* < 0.05) evaluated using a Student’s *t*-test. n represents sample size. Data are presented as mean and standard error.

To evade plant immunity, pathogens mutate the nucleotide sequences of their avirulence effectors, resulting in nonsynonymous substitutions. Therefore, we used BLAST to investigate whether there were sequence variations in *FocSIX5* among different isolates. Notably, a single-nucleotide polymorphism (G200A) was detected in the *FocSIX5* nucleotide sequence of the Foc_A21 strain isolated from the United Kingdom [8] and the Fox129 strain isolated from Finland [5], leading to a nonsynonymous substitution (R67K) (Figure 4a). Additionally, the same nonsynonymous substitution (R67K) was found in the Australian AP117 strain as in our *Foc* collection (Accession No. LC731005). Thus, we speculate that this nonsynonymous substitution may be a strategy used by *Foc* to avoid recognition by the host. To test this hypothesis, we generated a gene complementation mutant with the *FocSIX5* gene construct, including the G200A SNP (ΔSIX5-2 + SIX5^R67K^-1 [Δ-2 + SIX5^R67K^-1] and ΔSIX5-2 + SIX5^R67K^-2 [Δ-2 + SIX5^R67K^-2]) (Figure S5), and performed pathogenicity tests on shallots. Contrary to our hypothesis, shallot plants inoculated with mutants expressing SIX5 protein variants carrying the R67K substitution exhibited a highly resistant phenotype, suggesting that host plant immunity was not related to a single amino acid mutation (R67K) in FocSIX5 (Figure 4b,c).

## 4. Discussion

Plant-pathogenic fungi secrete effectors that manipulate the host immunity to induce infections. However, some effectors are recognized as avirulent by innate plant immune receptors, which cause robust plant resistance responses [15,40,41]. In *F. oxysporum*, avirulence effectors such as SIX1 (AVR3), SIX3 (AVR2), SIX5, and SIX4 (AVR1) in *Fol* and SIX6 in *F. oxysporum* f. sp. *niveum* (which infects watermelons) have been identified [22,23,42]. Among the SIX effectors in *Fol*, SIX5 is required for full virulence in susceptible tomato lines, and a SIX5 homolog is present in *Foc* [6,23]. Therefore, we investigated whether SIX5 in *Foc* is related to pathogenicity in onion and shallot plants, as has been reported for SIX5 in *Fol*-tomato pathosystem [23]. In the present study, we confirmed that *FocSIX5* in *Foc*_TA was expressed in both onion and shallot roots during the *Foc*-infection process. The *FocSIX5* gene was expressed in shallot and onion roots inoculated with *Foc*_TA (Figure S3), suggesting that *FocSIX5* may play an important role in the pathogenicity of *Foc* in onion and shallot infections. To investigate the relationship between the *FocSIX5* gene and pathogenicity during *Foc* infection in onions and shallots, we generated *FocSIX5* gene-modified mutants. Upon conducting a pathogenicity test, onion bulbs inoculated with the wild-type *Foc*_TA strain, *FocSIX5* knockout mutants, or *FocSIX5* complementation mutants exhibited typical symptoms of Fusarium basal rot disease, and the symptom areas were not considerably different between the wild-type *Foc*_TA and *FocSIX5* gene-modified mutants. However, the symptom area in onions inoculated with *FocSIX5* knockout mutants was slightly decreased compared to that in wild-type *Foc*_TA or *FocSIX5* complementation mutants. Given that *FocSIX5* gene knockout mutants showed the same colony-formation capability as wild-type *Foc*_TA and *FocSIX5* gene complementation mutants (Figure S5), the *FocSIX5* gene was specifically upregulated during *Foc* infection of susceptible onion, suggesting that FocSIX5 plays a role in the colonization of host plants rather than growth in onion. Similarly, SIX5 of *Fol* is related to virulence; thus, we speculate that *FocSIX5* gene is related to virulence in onions. Nevertheless, shallot plants inoculated with the *FocSIX5* knockout mutant showed more severe wilt symptoms than those inoculated with the wild-type *Foc*_TA, indicating that FocSIX5 secreted by *Foc* acts as an avirulence effector in shallots. Although the mechanism underlying the severe disease symptoms in shallot plants inoculated with the *FocSIX5* gene knockout mutants is not clear from the present study, it has been reported that host plants inoculated with avirulence gene knockout *F. oxysporum* mutants showed more severe wilting than those inoculated with wild-type *F. oxysporum* [22,23,42]. To the best of our knowledge, this is the first report of an avirulence effector in *Foc* toward *Allium* species.

This study confirmed that *FocSIX5* in *Foc*_TA was expressed in both onion and shallot roots during the *Foc*-infection process. The *FocSIX5* gene was expressed in shallot and onion roots inoculated with *Foc*_TA (Figure S3), suggesting that *FocSIX5* may play an important role in the pathogenicity of *Foc* in onion and shallot infections. To investigate the relationship between the *FocSIX5* gene and pathogenicity during *Foc* infection in onions and shallots, we generated *FocSIX5* gene-modified mutants. Upon conducting a pathogenicity test, onion bulbs inoculated with the wild-type *Foc*_TA strain, *FocSIX5* knockout mutants, or *FocSIX5* complementation mutants exhibited typical symptoms of Fusarium basal rot disease, and the symptom areas were not considerably different between the wild-type *Foc*_TA and *FocSIX5* gene-modified mutants. However, the symptom area in onions inoculated with *FocSIX5* knockout mutants was slightly decreased compared to that in wild-type *Foc*_TA or *FocSIX5* complementation mutants. Given that *FocSIX5* gene knockout mutants showed the same colony-formation capability as wild-type *Foc*_TA and *FocSIX5* gene complementation mutants (Figure S5), the *FocSIX5* gene is specifically upregulated during *Foc* infection of susceptible onion, suggesting that FocSIX5 plays a role in the colonization of host plants rather than growth in onion. Similarly, SIX5 of *Fol* is related to virulence; thus, we speculate that the *FocSIX5* gene is related to virulence in onions. Nevertheless, shallot plants inoculated with the *FocSIX5* knockout mutant showed more severe wilt symptoms than those inoculated with the wild-type *Foc*_TA, indicating that FocSIX5 secreted by *Foc* acts as an avirulence effector in shallots. To the best of our knowledge, this is the first report of an avirulence effector in *Foc* toward *Allium* species.

Pathogens undergo mutations in the avirulence effector that avert host recognition and promote infection [43,44]. The *Fol* race 3 strain has three different patterns of single amino acid substitutions in its AVR2 sequence (V41M, R45H, and R46P) to escape from *I-2*-derived immunity [22]. FocSIX5 is widely expressed in aggressive *Foc* [5,6,8]. Therefore, we speculate that there are variations in the FocSIX5 sequence. As expected, FocSIX5 had a single amino acid substitution, R67K, when sequence variants from different *Foc* strains were compared. To determine whether the R67K substitution affected avirulence, we complemented *FocSIX5* gene construct with an allelic variant carrying the G200A mutation. Shallot plants inoculated with mutants expressing SIX5 protein variants carrying the R67K substitution exhibited a highly resistant phenotype, suggesting that the single amino acid substitution R67K in FocSIX5 is not sufficient to overcome shallot resistance to *Foc*.

The SIX5 protein is conserved only within *Fol* and *Foc* in the *F. oxysporum* species complex with 74% identity and harbors cysteine residues at precisely the same positions. Interestingly, *AVR2* (*SIX3*) and *SIX5* share promoter regions in the *Fol* genome, and their encoded proteins physically interact with each other and are necessary for triggering *I-2*-derived immunity in tomato plants [23]. In *Foc*, *FocSIX3* and *FocSIX5* are located on the same scaffold, sharing both promoter regions with the reference genome of the *Foc*_FUS2 strain [28], and the nucleotide sequence of *FocSIX3* is 91.4% similar to that of *FolSIX3* [45]. Thus, FocSIX3-FocSIX5 may physically interact with each other and play a role similar to that of AVR2-SIX5, as reported by *Fol* [23]. However, shallot plants inoculated with the *FocSIX3* knockout mutant did not show the same wilt symptoms as those inoculated with wild-type *Foc*_TA, suggesting that FocSIX3 is not an avirulence effector recognized by the shallot, in contrast to the AVR2-SIX5 pair in *Fol*. Thus, it is possible that the putative immune receptor of shallots that recognizes FocSIX5 is unlikely to resemble but is partially similar to the I-2 receptor of tomatoes. The *I-2* gene encodes a nucleotide-binding and leucine-rich repeats (NB-LRR) at the N- and C-termini, respectively [25]. Further studies are required to explore the NB-LRR proteins that recognize SIX5 in shallots.

Some plant immune receptor proteins specifically interact with avirulent effectors secreted by the causative agents of the disease. For example, the immune receptor protein L6 in flax (*Linum usitatissimum*) interacts with the Avr567 avirulence protein in flax rust (*Melampsora lini*), causing a hypersensitivity reaction [46]. In addition, the resistance protein Pi-ta in rice and the avirulence effector AVR-Pi-ta in the rice blast fungus *Pyricularia oryzae* bind directly to each other to confer rice blast resistance [47]. *Foc*-resistance-related loci/genes in shallots are gradually being investigated; however, the specific loci and genes involved in disease resistance remain unclear [11,12]. Thus, FocSIX5 may be a useful tool for identifying *Foc*-resistant loci or genes in shallot. In this study, only one shallot genotype exhibiting high resistance to *Foc* was used. However, other shallot genotypes resistant to *Foc* have also been reported [48]. Hence, comparative genome analysis of these shallot genotypes and those used in this study could reveal *Foc*-resistant loci or genes, leading to an understanding of the resistance mechanism of shallots to *Foc* and the acquisition of a promising breeding resource for onion disease resistance.

Collectively, our results indicate that the high-*Foc*-resistance shallot cv. Chiang Mai and shallot immunity-recognizing FocSIX5 are promising breeding resources for disease resistance in onions against *Foc*.

## 5. Conclusions

Understanding the relationship between pathogens and plants is important for breeding disease-resistant varieties. In the present study, we demonstrated that FocSIX5 acts as an avirulent effector of *Foc* in shallots. Moreover, we demonstrated that a single amino acid substitution (R67K) in the FocSIX5 sequence was not associated with the ability to overcome shallot resistance. The insights gained from this study could be useful for the development of onion cultivars that are resistant to *Foc*.

## Figures and Tables

**Table 1:** Primers used in this study.

Primer Name	Sequence (5′–3′)	Purpose	Reference
SIX5-C-F	GCGCTTCGAGTACATCTCTG	Detection of *FocSIX5*	This study
SIX5-C-R	CTAGGATGCATCACAATAGA	Detection of *FocSIX5*	This study
SIX5-Q-F	TGCCACCACTCAGCTTCAGA	Quantification of *FocSIX5*	This study
SIX5-Q-R	TGAAATGTGGACCAAGTGCTCTA	Quantification of *FocSIX5*	This study
SIX5-split-F1	GGGATAGGTAAGCAAGCAGCTTG	Disruption and complementation of *FocSIX5*	This study
SIX5-split-F2	GTCGTGACTGGGAAAACCCTGG CGGTGATGAAGAGTAGTAGAG	Disruption of *FocSIX5*	This study
SIX5-split-F3	TCCTGTGTGAAATTGTTATCCG CTTCTGTCATTGTGACCAGTG	Disruption of *FocSIX5* Verification of *FocSIX5* gene knockout	This study
SIX5-split-F4	ATGTCAAGAGCGCGCGAAGCTC	Disruption and complementation of *FocSIX5* Verification of *FocSIX5* gene knockout	This study
FoTEF-Q2-F	CATCGGCCACGTCGACTCT	Quantification of *EF-1α*	[34]
FoTEF-Q2-R	AGAACCCAGGCGTACTTGAA	Quantification of *EF-1α*	[34]
M13F	CGCCAGGGTTTTCCCAGTCACGAC	Creation of *hph* construct	[35]
M13R	AGCGGATAACAATTCACACAGGA	Creation of *hph* construct	[35]

## Data Availability

Sequence data for FocSIX5 from the TA and AP117 strains were deposited in the DNA Data Bank of Japan under accession numbers LC730887 and LC731005, respectively.

## Supplementary Materials

The following supporting information can be downloaded from https://www.mdpi.com/article/10.3390/microorganisms11122861/s1. Figure S1: Relative gene expression of *FocSIX5* in *Foc*_TA during infection in onion and shallot; Figure S2: Schematic of the marker-exchange homologous recombination between *FocSIX5* gene and the hygromycin B resistant (*hph*) cassette; Figure S3: Verification of *FocSIX5* knockout mutant through Southern blotting analysis; Figure S4: Confirmation of transformation via polymerase chain reaction (PCR) in mutants with modifications in the *FocSIX5* gene; Figure S5: Confirmation of colony-formation capability in *FocSIX5* gene-modified mutants; Figure S6: Results of the pathogenicity test toward onion bulb with *FocSIX5* gene knockout and gene complementation mutant; Figure S7: Biomass of shallot plant inoculated with wild-type *Foc*_TA, *SIX5* gene knockout, and complementation mutants.
