# Supplementary material for: The SIX5 Protein in Fusarium oxysporum f. sp. cepae Acts as an Avirulence Effector toward Shallot (Allium cepa L. Aggregatum Group)
Source: Microorganisms. 2023 Nov 26;11(12):2861. doi: 10.3390/microorganisms11122861 (PMC10745378; doi:10.3390/microorganisms11122861)
Supplement: Supplementary file 1 [file microorganisms-11-02861-s001.zip › microorganisms-2660675-supplementary.pdf]

Supplementary Materials

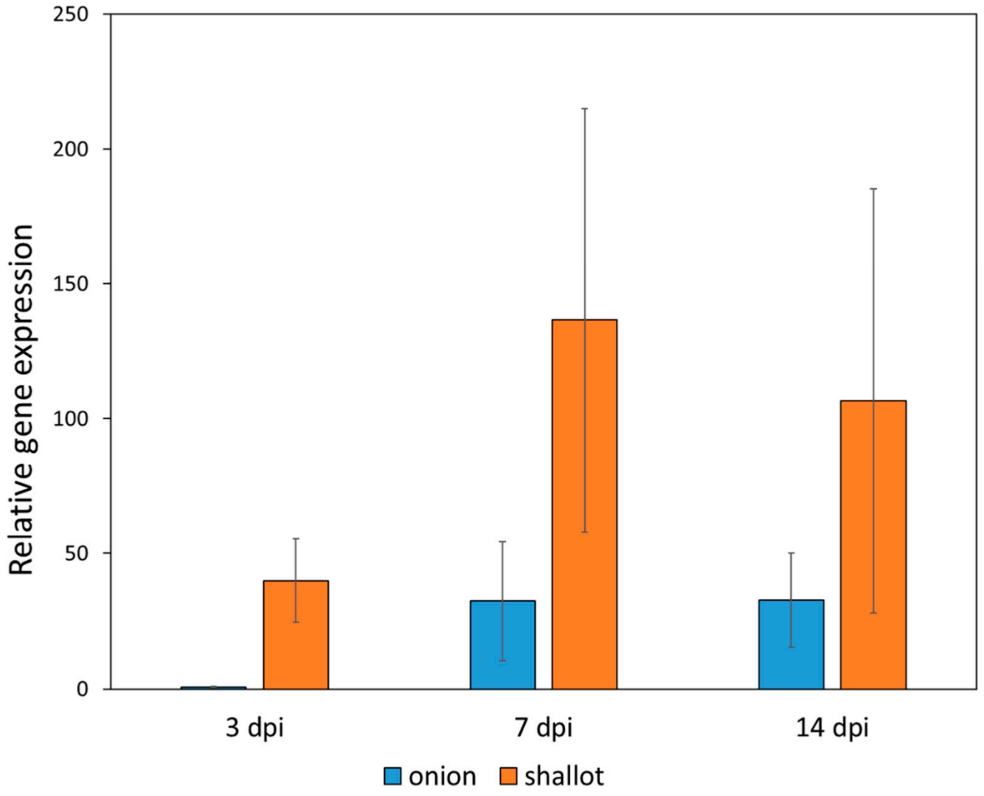

Figure S1. Relative gene expression of *FocSIX5* in *Foc*_TA during infection in onion and shallot. mRNA expression levels of *FocSIX5* genes at 3, 7, and 14 days post-inoculation (dpi) were determined using qRT-PCR. Relative amounts of transcripts of *FocSIX5* gene were calculated and normalized to that of *EF-1α* gene. Data are presented as mean and standard error (n = 3).

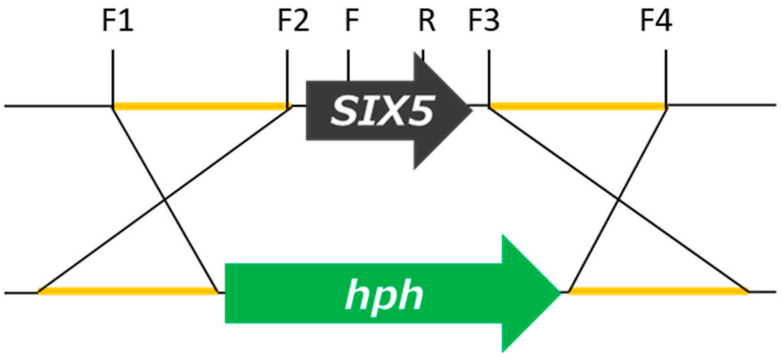

Figure S2. Schematic of the marker-exchange homologous recombination between *FocSIX5* gene and the hygromycin B resistant (*hph*) cassette. Yellow bars indicate identical upstream/downstream sequences for homologous recombination. The vertical solid lines illustrate each primer sites.

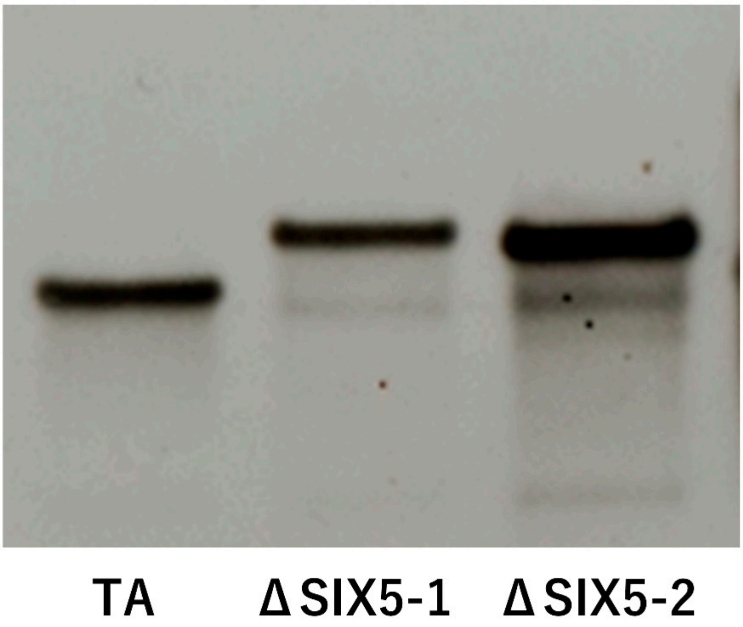

Figure S3. Verification of *FocSIX5* knockout mutant by Southern blotting analysis. Total DNA (10 μg) of wild-type *Foc*_TA and candidates of *FocSIX5* gene knockout mutants was digested using *Eco*RV restriction enzyme, and after blotting, hybridized using hybridization probe**.** TA, ΔSIX5-1, and ΔSIX5-2 lane indicates the result of southern blotting of each strain, respectively.

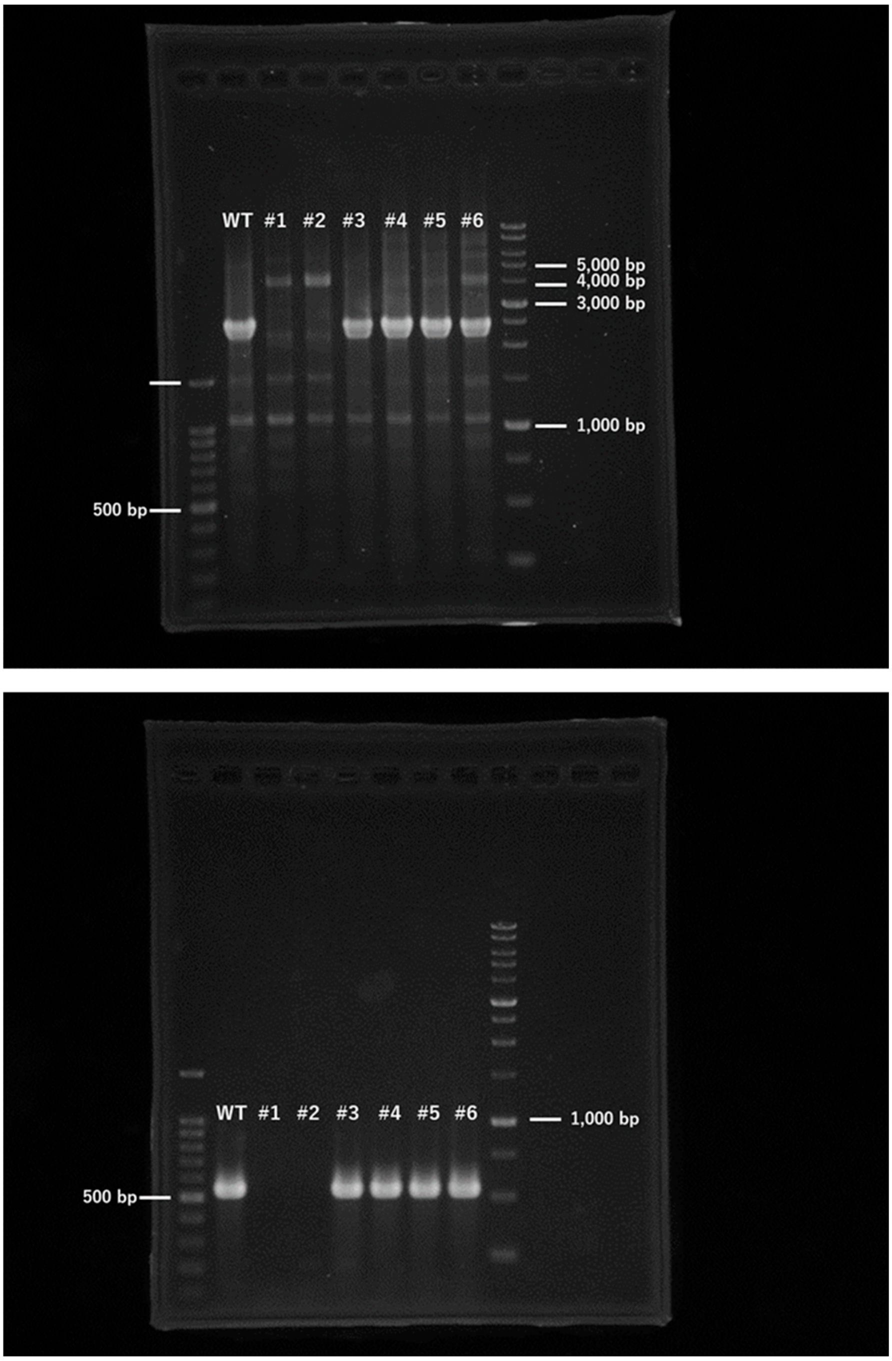

Figure S4. Confirmation of transformation by polymerase chain reaction (PCR) in mutants with modifications in the *FocSIX5* gene. The upper and lower panels show PCR amplification of upstream and downstream region, including *SIX5* gene with a SIX5-split-F1–SIX5-split-F4 primer set (upper panel) and *SIX5* gene coding region with the SIX5-C-F- SIX5-C-R primer set (lower panel). WT: *Foc*_TA. #1: ΔSIX5-1. #2: ΔSIX5-2. #3: ΔSIX5-1+SIX5. #4: ΔSIX5-2+SIX5 #5: ΔSIX5-2 + $SIX5^{R67K}$-1. #6: ΔSIX5-2 + $SIX5^{R67K}$-2.

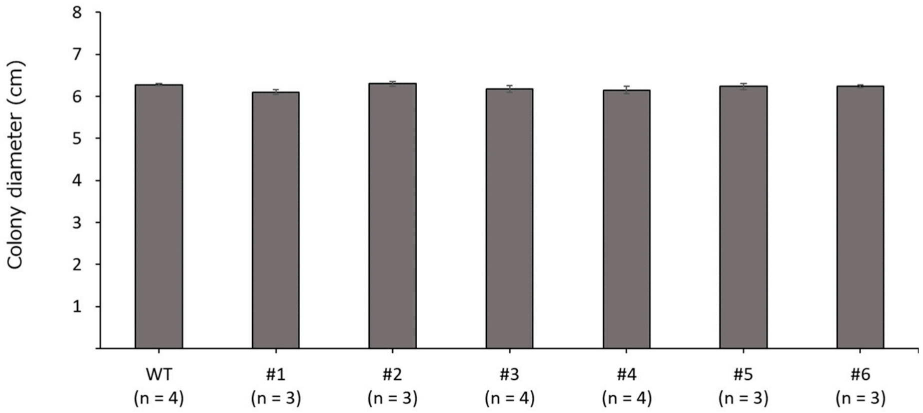

Figure S5. Confirmation of colony formation capability in *FocSIX5* gene-modified mutants. The statistically significant difference at $p < 0.05$ were analyzed by one-way ANOVA post hoc Tukey HSD test. n represents sample size. All data were presented as mean and standard error. WT: *Foc*_TA. #1: ΔSIX5-1. #2: ΔSIX5-2. #3: ΔSIX5-1+SIX5. #4: ΔSIX5-2+SIX5 #5: ΔSIX5-2 + $SIX5^{R67K}$-1. #6: ΔSIX5-2 + $SIX5^{R67K}$-2.

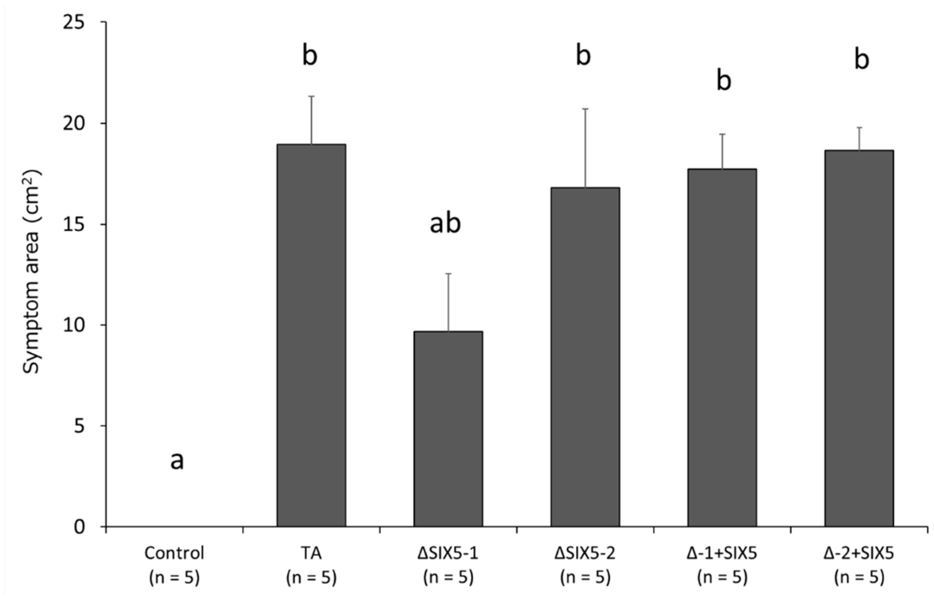

Figure S6. Results of the pathogenicity test toward onion bulb with *FocSIX5* gene knockout and gene complementation mutant. Symptom area on onion bulb inoculated with wild-type *Foc*_TA, *FocSIX5* gene knockout (ΔSIX5-1 and ΔSIX5-2), and *FocSIX5* gene complementation mutants (ΔSIX5-1 + SIX5 [Δ-1 + SIX5] and ΔSIX5-2 + SIX5 [Δ-2 + SIX5]) four weeks after inoculation. The statistically significant difference at $p < 0.05$ were analyzed by one-way ANOVA post-hoc Tukey HSD test. n represents sample size. All data are presented as mean and standard error.

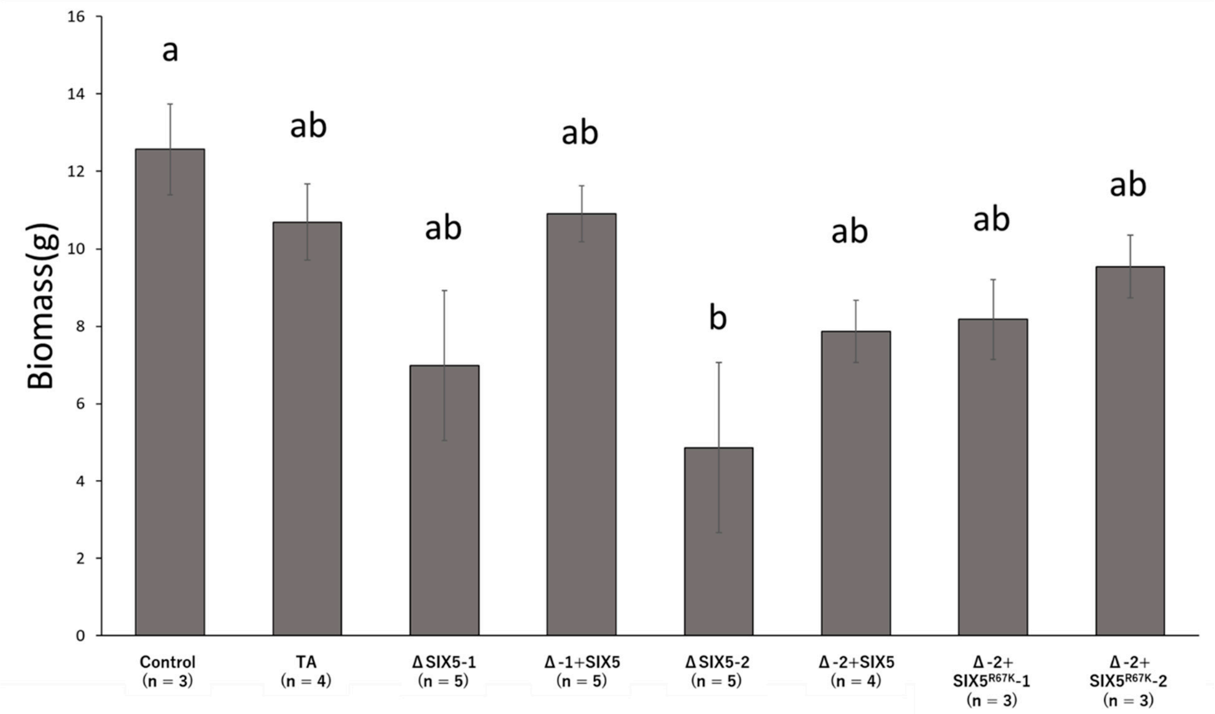

Figure S7. Biomass of shallot plant inoculated with wild-type *Foc*_TA, *SIX5* gene knockout, and complementation mutants. Average biomass of shallot plants inoculated with wild-type *Foc*_TA, *FocSIX5* gene knockout (ΔSIX5-1 and ΔSIX5-2) and gene complementation mutant (ΔSIX5-1 + SIX5 [Δ-1 + SIX5], ΔSIX5-2 + SIX5 [Δ-2 + SIX5], ΔSIX5-2 + $SIX5^{R67K}$-1 [Δ-2+ $SIX5^{R67K}$-1], and ΔSIX5-2+ $SIX5^{R67K}$-2 [Δ-2+ $SIX5^{R67K}$-2]) with five weeks after inoculation. The statistically significant difference at $p < 0.05$ was analyzed by one-way ANOVA post hoc Tukey HSD test. n represents sample size. Data are presented as mean and standard error.
